# Functional outcome of flexor tendon repair of the hand at Zone 5 and post operative early mobilization of the fingers

**DOI:** 10.12669/pjms.291.2563

**Published:** 2013

**Authors:** Seyed Abdolhossein Mehdi Nasab, Nasser Sarrafan, Seyed Reza Saeidian, Hassan Emami

**Affiliations:** 1 Seyed Abdolhossein Mehdi Nasab, Associate Professor, Department of Orthopaedic Surgery, Emam Khomeini Hospital, Musculoskeletal and Rehabilitation Research Center, Jundishapur University of Medical Sciences, Ahvaz, Iran.; 2 Nasser Sarrafan, Associate Professor, Department of Orthopaedic Surgery, Emam Khomeini Hospital, Musculoskeletal and Rehabilitation Research Center, Jundishapur University of Medical Sciences, Ahvaz, Iran.; 3 Seyed Reza Saeidian, Assistant Professor, Dept. of Physical Medicine & Rehabilitation, Emam Khomeini Hospital, Musculoskeletal and Rehabilitation Research Center, Jundishapur University of Medical Sciences, Ahvaz, Iran.; 4 Hassan Emami, Resident, Department of Orthopaedic Surgery, Emam Khomeini Hospital, Musculoskeletal and Rehabilitation Research Center, Jundishapur University of Medical Sciences, Ahvaz, Iran.

**Keywords:** Flexor Tendon Injury, Hand Function, Kessler Technique, Early Finger Mobilization, Zone 5

## Abstract

***Objective*** *:* There are few reports on outcome following flexor tendon repair of the hand in zone 5. We hypothesized that early mobilization of the fingers is possible if the suture site of repaired tendon is strong enough. The aim of this study was to assess the results of flexor tendon repair in this zone using modified Kessler method reinforced by peripheral running suture and a post operative early active and passive mobilization of the fingers.

***Methodology:*** This prospective study was carried out between April 2006 and Feb 2010, and 171 digits flexor tendons cut in 42 patients were repaired by modified Kessler technique reinforced by running peripheral suture**.** Early active mobilization and gentle passive motion of the fingers was allowed in a dorsal wrist splint the day after surgery. Wrist Immobilization was performed for one month. Function of the tendons was assessed by Buck-Gramcko score at nine month follow up.

***Results:*** Mean age of the patients was 25.4 years (range 17-46 y). Twenty nine flexor policis longus, 77 flexor digitorum superficialis and 65 flexor digitorum profundus tendons of digits were repaired. Middle and index fingers were most commonly involved. Median and ulnar nerve repair was done in 17 and 12 cases respectively. Good to excellent results were seen in of 79.34% of FPL and 74.65% of other finger flexors. One case of FPL rupture was seen. Tenolysis of FDS was performed in one case. Recovery in thenar muscle function was good, fair and poor in 5, 2 and 10 cases after median nerve repair, while all 12 patients with ulnar nerve lesion showed some degrees of clawing of 4^th^ and 5^th^ fingers.

***Conclusion*** *:* Most patients following flexor tendon repair at zone 5 obtained good results. Early motion of the fingers seems to improve outcome in these patients. Concomitant nerve cut in particular of ulnar nerve were associated with a high rate of poor results.

## Introduction

 Hand is one of the most active parts of the body, and its normal function is essential for daily activities. Function of the hand and fingers is related to normal integrity of the bones, tendons and neurovascular structures, and injuries in any parts of these organs can deteriorate the hand function. Flexor tendons of the hand are considered in five anatomic zones due to peculiarities of each area. Zone V extends from the proximal border of the transverse carpal ligament to the musculo tendinous junction in the proximal part of the forearm.^1^^,^^2^ Flexor tendon lacerations in the forearm are frequently associated with laceration of the nerve and artery which further compromise the function of the hand.

 Despite many surgical techniques and appropriate rehabilitation programs, flexor tendon injuries may be associated with adhesion formation and loss of hand function.^3^^,^^4^ Primary surgical repair with restoration of length, strength and gliding excursion of tendon cut is essential and primary repair would have the best outcome. Results of flexor tendon cut after repair depends on many factors such as concomitant nerve injury, technique and type of repair, surgeon’s experience, nature of the lesion, and post operative rehabilitation. Though this injury is frequently seen at our emergency department, but no studies have been done to assess their functional outcome in our region. On the other hand there is little in the literature to indicate the best protocol of post operative rehabilitation after flexor tendon repair in zone 5.^5^^,^^6^^,^^7^

 The aim of this study was to evaluate the results of flexor tendon laceration repair in zone 5 treated with early motion of the digits after surgery.

## Methodology

 This prospective study was carried out from Apr 2006 to Feb 2010 in orthopedic departments of Emam Khomeini and Arvand hospitals in Ahvaz, Iran, and all the consecutive patients with flexor tendon lacerations due to sharp cuts were included. Patients younger than 16 years, severe crushing injury, associated fractures of the hand or forearm and non compliant patients during follow up were excluded. Also we excluded results of wrist flexor tendons (FCR, FCU, and PL) from the study. Forty two patients with 171 digits flexor tendon injury were admitted. Surgical repair was performed under general anesthesia in 40 patients and local double tourniquet in two patients. Follow-up was performed for 9 month (6-13 months). Repair was done by modified Kessler technique using 4-0 monofilament nylon, reinforced by continuous running suture. We assessed stability of the repair by gentle passive motion before closure of the wound. Vascular injuries of radial or ulnar were repaired or ligated according to the condition of the wound and time since injury, and any cut in median or ulnar nerve was repaired by a 6-0 or7-0 monofilament nylon stitches. After closure of the wound, they were placed in a dorsal cast splint with the wrist in 40-50 deg of flexion, metacarpophalangeal joints in 30-40 deg of flexion. Patients were encouraged to do active and passive movement in the splint the day after surgery. By four weeks the splint and stiches were removed and more active motion was encouraged. Two weeks after splint removal, patients were referred for physiotherapy.

**Table-I:** Buck-Gramcko scoring system.

*Criteria*	*Units*	*Points*
Distance between fingertip and distal palmar crease(cm) composite flexion(deg)	0-2.5>200	6
	2.5-4>180	4
	4-6/>150	2
	>6/<150	0
Extension deficit (deg)	0-30	3
	31-50	2
	51-70	1
	>70	0
Composite flexion minus composite extension(deg)	>160	6
	>140	4
	>120	2
	>120	0
Evaluation	Excellent	14-15
	Good	11-13
	Fair	7-10
	Poor	0-6

 Follow-up visits was performed after splint removal then at 3, 6, 9 and 12 month post surgery. At final flow-up the outcome were assessed by Buck-Gramcko score.^8^ (Table-I) This study was approved by ethics committee at our university, and an informed consent was taken as a routine approach at the time of admission. Statistical analysis was performed using t test in SPSS software version 13 and significant difference was considered when the p.value was ‹0.05.

## Results

 Forty two patients with a total of 171 digits flexor tendon cut at zone 5 were repaired. (Table-II). There were 40 men and two women, with an average age of 25.4 years (17-46 years). The most frequent cause was by glass injury in 25, knife in 15 and a sharp metal instrument in two patients. The average tendon injury in each patient was 4.1. In addition to tendon cutting, median and ulnar nerve cut were seen 17 and 12 patients respectively. Ulnar and radial artery laceration was present in 10 and 7 patients respectively. In the patients with cuts in both arteries, repair was done by a general or vascular surgeon, while in the case of single artery laceration, repair or ligation was done according to the condition of the wound and time since injury.

**Table-II:** Details of flexor tendon injuries in Zone 5.

*FDP*	*FDS*	*Number of tendon repair*	*Fingers *
-	-	29	Thumb (FPL)
16	22	38	Index
18	22	40	Middle
17	19	36	Ring
14	14	28	Little
65	77	171	Total

FDS= Flexor digitorum profundus, FDS= flexor digitorum superficialis

**Table-III:** Functional results of patients according to the B.G score.

*Tendons *	*Excellent*	*Good*	*Fair*	*Poor*
FPL thumb, N=29	N=16 (55.2%)	N= 7(24.14%)	N= 2(6.90%)	N= 4(13.80%)
Fingers flexor tendons, N=142	N= 76 (53.52%)	N=30(21.13%)	N=12 (8.46%	N= 24(16.88%)
Total= 171	92 (54.36%)	37(22.63%)	14(7.68)	28(15.33%)

 Right hand was involved in 25 and left hand in 17 patients. Laceration in tendons of the middle and index fingers was the most common injuries. Rupture of FPL tendon was noted in one patient and tenolysis of FDS was needed in one patient (2.3%). Poor results were seen in 4 cases (13.80%), after FPL repair and in 24 (16.90%) after fingers. According to the Buck-Gramko score the maximum score for thumb and other fingers were 13 and 14 respectively. Good and excellent results for the thumb and fingers were seen in 79.34% and 74.65% of the fingers. (Table-III). Median and ulnar nerve repair was done in 17 and 12 cases respectively. All cases with ulnar nerve involvement developed some degrees of claw hand deformity. Atrophy in thenar muscles was seen in 10 out of 17 patients with median nerve injury. Sensory deficit as two- point discrimination in average of 12 mm was present in all patients. With respect of motor recovery in the hand intrinsic muscles, good results in patients sustained a nerve injury was seen in 29.5% of patients.

## Discussion

 Hand is one of the most active parts of the upper extremity but with less protection. The dexterity of hand in various professional, psychological and social activities exposes it to a multitude of dangers, occupational as well as social. As a result the injuries of the hand are commonly seen young working class. Results of flexor tendon repair depends on many factors such as time since injury, technique of suture, associated injuries, surgeon’s experience, and post operative rehabilitation. There are many post operative mobilization protocols ranges from strict immobilization to early protected mobilization of the fingers.^9^ The most frequent mechanism of injury was by broken glass and knife in 95.2% of the cases which are similar to previous reports. In the review of literature only a few reports were found with respect of flexor tendon repair in zone 5 and much of the works are on results of tendon repair in zone 2. Stefanich et al on their study on 32 patients with zone V flexor tendon laceration rehabilitated by the Kleinert protocol showed good to excellent results in 88% of the patients.^10^ Hung LK and Pang KW studied 32 patients with flexor tendon injuries repaired by the modified Kessler method. With a dorsal plaster slab and early active motion of the fingers good to excellent results was achieved in 77% of their cases^11^

 Olivier et al compared two technique of motion - stable wire suture (MSWST) and modified Kessler suture for flexor tendon repair. In MSWST group splint was not applied and early motion was allowed, but outcome was similar in both group.^12^ Hudson and Dejager reported the results of 76 flexor tendon repair in 15 patients and found 36 excellent, 5 good, and 20 fair and 15 poor results in the patients.^13^ Kabak et al in their series of 21 patients with simultaneous median or ulnar nerve lacerations with flexor tendons cuts, found 76% good to excellent results and better outcome for median nerve than ulnar nerve repair.^14^ In our study return of thenar muscle function was better than intrinsic ulnar muscles innervated. Wilhelmi et al reviewed 168 zone V finger flexor tendons repair for 29 pateintsand used a protocol of early active motion postsurgery.they achieved good to excellent functional in 85% of the casas.^15^ In a study by Yii et al, good to excellent results were achieved in 90% of flexor tendon repair in zone 5.^16^ Cigdem B et al studied outcome of 15 patients with repaired zone V flexor tendon injuries using a post operative rehabilitation program of modified Kleinert and Duran. They reported 92.8% of the fingers achieved excellent results.^9^ The reason of more excellent rates in these studies may be the fact that only the function of the digits tendons have been considered. As in our study, combined injuries in tendons, artery or nerves are associated with deteriorate hand function, which led to some permanent disability. 

 In our study good and excellent results were obtained in 74.65% of flexor tendons of digits. Complications after tendon repair may be rupture or adhesive formation. Despite allowing early active motion in splint and after removal of it, only in 1 (0.59%) patient, flexor pollicis tendon rupture was noted 8 weeks after repair. Stefanish et al reported 2 tendon rupture.^10^ In a study by Saini et al rupture occurred in 3% of their casas.^7^ Adhesion formation occurred in 2 FDS tendons in one of our patients (1.17%). This low rate of complications could be due to meticulous and strong tendon repair in particular of continues peripheral running suture technique and also, early mobilization of the fingers in our patients.

 It is clear that simultaneous injury in median or ulnar nerve with flexor tendon cuts will further deteriorate the functional outcome with respect of grip strength and pinch strength of the hand, but isolated median or ulnar nerve injuries does not affect the overall gliding excursion of the repaired tendons^7^ In our series, 29 (69%) patients had median and or ulnar nerve laceration. Recovery in intrinsic muscles in this group was achieved in 29.5% of cases. In 13 (31%) other patients who had no neurovascular injuries, excellent to good results in motion of fingers was seen in 74.65% repaired tendons. Poor results in our patients were seen mostly in cases that had nerve cut, in particular with ulnar nerve. However for assessment of median or ulnar nerve repair separate studies will be needed. This may be attributed to paralysis and atrophy of intrinsic hand muscles. Nerve repair using binocular loop or microscope may improve outcome in these patients. We did not use these instruments for nerve repair, which may be a limitation in our study.

***Limitations of study:*** We did not include wrist flexor tendons (FCR, FCU.PL) repair in our study, because with Buck-Gramko score, measurement of wrist flexor tendons cannot be evaluated. Also, for assessment of grip strength, pinch strength, and subjective hand function of median or ulnar nerve repair in this zone, further study will be needed.

 We conclude that modified Kessler technique reinforced with continues running suture can provide enough strength at flexor tendon repair site to permit early gentle passive and active motion of the fingers. The rate of tendon rupture or adhesion formation with this protocol was low. Thus strong repair and early mobilization of the fingers after flexor tendon repair are key factors to obtain good results and to prevent adhesive formation. Patients with associated neurovascular laceration in particular with ulnar nerve cut, showed poor results. We strongly advise early mobilization of the fingers following their tendon repair in zone 5.
